# Rhabdomyolysis as Potential Late Complication Associated with COVID-19

**DOI:** 10.3201/eid2609.201463

**Published:** 2020-09

**Authors:** Ying-Chao He, Feng Chen

**Affiliations:** Fujian Provincial Hospital, Fuzhou, Fujian, China

**Keywords:** COVID-19, rhabdomyolysis, respiratory infections, severe acute respiratory syndrome coronavirus 2, SARS-CoV-2, SARS, 2019 coronavirus disease, zoonoses, viruses, coronavirus

**To the Editor:** We provide follow-up information on a case discussed in Emerging Infectious Diseases of a man with severe acute respiratory syndrome coronavirus 2 (SARS-CoV-2) infection who reportedly had late-onset rhabdomyolysis with lower limb pain and fatigue (*1*). After the patient was stabilized, he was transferred to Wuhan Union Hospital, where he disclosed symmetric weakness (Medical Research Council grade 4/5) in both lower limbs with weakened deep tendon reflexes and decreased sensation to light touch and pinprick distally. Because weakness and paresthesia persisted after biochemistries normalized, we feel that these observations are not explained solely by rhabdomyolysis.

The patient was discharged 43 days after admission and was able to walk normally but with reduced endurance. Electromyography (day 120) showed motor and sensory fiber involvement in both lower extremities, presenting as axonal injury accompanied by demyelination (Tables 1, 2). Despite his 10-year history of diabetes, the patient reported no history of paresthesia or reduced motor endurance, which ruled against preexisting diabetic neuropathy or myopathy. We believe he developed peripheral neuropathy during his COVID-19 illness, which may have been missed during the acute phase. We are unsure what caused this neuropathy. In addition to hematologic or lymphatic spread, coronaviruses may directly invade the peripheral nerve terminals and interfere with subsequent synaptic transfer (*2*). Indirect causes, such as cytokine-mediated damage, should also be considered in this patient (*3*). Finally, thromboembolism has the potential to cause peripheral nerve ischemia and necrosis (*4*). However, the coagulation indices, including fibrinogen (7.95 g/L, reference 2–4 g/L), d-dimer (>20 mg/L, reference <0.5mg/L), and fibrinogen degradation products (80 μg/mL, reference <5 μg/mL), were at the highest level at the onset of rhabdomyolysis and gradually decreased with enoxaparin treatment. We cannot offer a definitive diagnosis and were limited by the lack of muscle biopsies and complete electromyography; however, several factors may have caused his peripheral neuropathy.

**Table 1:** Motor nerve conduction studies on a patient with rhabdomyolosis after severe acute respiratory syndrome coronavirus 2 infection, China

Location	Distal latency, ms	Amplitude, mV	Conduction velocity, m/s	F latency, ms
Left tibial nerve				
Ankle-abductor hallucis brevis	6.5 (reference <5.1)	0.825 (reference >4)	38 (reference >40)	51.4 (reference <56)
Popliteal fossa	15.4	0.755		
Right tibial nerve				
Ankle-abductor hallucis brevis	6.3 (reference <5.1)	5.4 (reference >4)	39 (reference >40)	49.4 (reference <56)
Popliteal fossa	15.0	4.46		
Left peroneal nerve				
Ankle-extensor digitorum brevis	5.1 (reference <5.5)	1.061 (reference >2)	35 (reference >42)	Not tested
Below fibula	11.6	1.022		
Right peroneal nerve				
Ankle-extensor digitorum brevis	3.8 (reference <5.5)	1.947 (reference >2)	33 (reference >42)	Not tested
Below fibula	10.7	1.328		

**Table 2:** Antidromic sensory nerve conduction studies on a patient with rhabdomyolosis after severe acute respiratory syndrome coronavirus 2 infection, China

Location	Amplitude, μV	Conduction velocity, m/s
Left superficial fibular nerve: lateral calf–lateral ankle	Absent	Absent
Right superficial fibular nerve: lateral calf–lateral ankle	5.879 (reference ≥6)	37 (reference ≥40)
Left sural nerve: calf–posterior ankle	Absent	Absent
Right sural nerve: calf–posterior ankle	4.225 (reference ≥6)	37 (reference ≥40)

## References

[R1] Jin M, Tong Q. Rhabdomyolysis as potential late complication associated with COVID-19. Emerg Infect Dis. 2020;26:1618–20. 10.3201/eid2607.200445 32197060 PMC7323559

